# A Higher Professional Standard

**Published:** 1892-03

**Authors:** 


### A Higher Professional Standard.

Much has been said, of late, as to the necessity of elevating the standard of medical education. The work and results of medical colleges have been scrutinized and criticised, and laws passed and reformation advocated to raise the average college course, and regulate the routine of study.

All this is very well, and we are glad indeed that the best medical colleges in the land have responded to the demand. Our young men will be better fitted for their work, and the increased difficulty of entering the profession will prevent the old men from being crowded out by the great army of new-comers.

To stop short at the medical colleges, however, is far from right. When we see growing tendencies to loose and irregular practice among those who know better, and the strife and jealousy which so often exists among men whose education and work should make them brothers, we feel that there is an equal demand for a higher professional standard among those who are practicing medicine, as those who are at the threshold as students.

We have heard a man insist upon a higher grade course of study for students, and closer examination for graduates, while it was an open secret that his own methods were questionable and his personal character rotten. Indeed, it is often such fellows who make the most noise and, as a consequence, excite the most disgust.

Let the work of reformation which has begun in the schools, be carried on in the profession, until our ranks are free from those who disgrace themselves and all connected with them. There are enough of honest, right-minded men in the profession to freeze out or reform all these fellows—half doctor and half quack. What we need is a recognition of a man's true standard, and the courage to treat him according to our convictions and his deserts.—*St. Louis Clinique.*

---

The above applies quite as well to dental colleges and dental education, as to those pertaining to general medicine. Improvement is undoubtedly being made, but is tardy in its coming. May the day hasten on when there shall be a higher appreciation of the importance of this subject, and when this will result in actual experience and practical demonstration.—ED. DENTAL REGISTER.

